# Damage index in childhood-onset systemic lupus erythematosus in Egypt

**DOI:** 10.1186/1546-0096-9-36

**Published:** 2011-12-09

**Authors:** Samia Salah, Hala M Lotfy, Abir N Mokbel, Ahmed M Kaddah, Nouran Fahmy

**Affiliations:** 1 Department of Pediatrics Faculty of Medicine, Cairo University; 2 Department of Rheumatology Faculty of Medicine, Cairo University

**Keywords:** Organ damage, systemic lupus erythematosus, Egyptian children

## Abstract

**Background:**

To investigate the prevalence of cumulative organ damage among Egyptian children with juvenile-onset systemic lupus erythematosus (jSLE) and the relationships between the organ damage and the demographic data, clinical variables, and disease activity.

**Methods:**

A total of 148 patients with jSLE have been followed in the pediatric rheumatology clinic and section at Cairo University. These patients were evaluated by retrospective chart review. The organ system damage due to SLE was measured using the Systemic Lupus International Collaborating Clinics/American College of Rheumatology Damage Index (SDI). Risk factors for damage were also studied including demographic criteria as well as clinical and laboratory manifestations.

**Results:**

Overall, 43.9% of the patients had damage within a mean of 6.57 ± 3.59 years of disease diagnosis. Neuropsychiatric (NPS-21%) and renal (16.9%) system involvement were observed most frequently, followed by cardiovascular (11.5%), skin (9.5%), pulmonary (6.1%), and ocular (4.8%), with a mean SDI score of 0.93 ± 1.37. In our study, the presence of neuropsychiatric manifestations at diagnosis showed the strongest association with the presence of later disease damage.

The number of SLE diagnostic criteria at presentation was strongly associated with the total SDI score, and the renal damage was significantly more prevalent in patients with age at disease diagnosis below 10 years of age. A higher mean disease duration was found in patients with musculoskeletal damage.

**Conclusion:**

We found that cumulative organ damage, as measured by the SDI, was present in 43.9% of Egyptian patients with juvenile-onset SLE. The damage was significantly more likely in patients who had more SLE diagnostic criteria at time of disease presentation and NPS manifestations at the time of diagnosis.

## Background

Systemic lupus erythematosus (SLE) is a chronic autoimmune disease characterized by autoantibodies directed against nuclear antigens and causing a variety of clinical and laboratory abnormalities [1]. SLE may involve multiple organs, causing significant morbidity and mortality in adults, adolescents, and children [2].

Over the last few decades, there has been a remarkable improvement of survival among patients with juvenile-onset systemic lupus erythematosus (jSLE). Case series from the 1980s and 1990s document that 83-93% of patients survive for 5 years [3] with some authors reporting 76-85% of patients alive at 10 years [4]. The improved survival may be due to earlier diagnosis and better approaches to treatment [5]. Other factors such as improved intensive care unit medical care and other supportive services may have a role. As a result, children and adolescents with jSLE are now faced with considerable morbidity due to the sequelae of disease activity, side effects of medications, and comorbid conditions [6]. This morbidity may affect their long-term quality of life, leading to problems related to the physical and psychological adaptation to a chronic severe illness. The management of patients with jSLE is now directed not only at preventing death, but also at lessening the development of permanent damage to involved organ systems resulting from the disease, its therapy, or disease complications [6].

The Systemic Lupus International Collaborating Clinics/American College of Rheumatology Damage Index (SDI) [7] is an instrument that includes assessment of 12 body organ systems and helps document irreversible damage occurring in patients with lupus. The objective of the present study is to investigate the frequency of accumulated organ damage in Egyptian patients with jSLE and to assess its association with demographic, clinical factors, and disease activity.

## Methods

The present study included 148 Egyptian patients with jSLE all fulfilling the inclusion criteria of this work, which were as follows: (1) Each patient having four or more of the 1997 revised American Rheumatism Association SLE criteria at the time of assessment of damage [8]; (2) Each patient age at disease diagnosis at or below 16 years; and (3) All patients having a disease duration of 6 months at study entry.

All patients were recruited from, and were followed up at, the Pediatric Rheumatology clinic of Cairo University Children's Hospital in Cairo, Egypt from January 1990 to December 2008. Initial data at the time of first presentation were obtained for all patients using chart review. The follow-up data extracted from the files were used to confirm the presence of damage at that point of damage assessment. Patients who died were excluded as well as loss-to-follow-up patients who failed to attend the clinic for more than 12 months. Patients whose follow-up was less than one year were also excluded from the study.

For all patients, demographic and clinical information were reviewed including: 1) Sex; 2) Age of disease diagnosis; 3) Age at time of assessment; 4) Disease duration from diagnosis to study visit; 5) Disease manifestations at presentation; and 5) Clinical and laboratory data that might represent risk factors for the development of permanent damage.

The time of disease presentation was defined as the time of development of the first ACR SLE criterion. Lupus activity was assessed by the SLE Disease Activity Index (SLEDAI) [9]. The accumulated damage was measured by the SDI [7]. Briefly, the SDI is a validated physician-rated instrument consisting of 41 items in 12 organ systems/domains ascertained by clinical assessment and has a possible range of 0-49. Damage is defined as any nonreversible change, not related to active inflammation, occurring since onset of SLE, detected by clinical assessment, and present for at least 6 months. Damage may be due to the disease itself, its treatment, or comorbid conditions [7]. As growth failure represents a significant source of damage in the pediatric age that is not included in the SDI, we also recorded its occurrence. Growth failure was defined as a height below the 5th percentile for age or a decreased growth that crossed 2 major percentiles [10]. Growth assessment was based on Egyptian growth curves [11]. Assessment of delayed puberty was also important for evaluation of damage in patients with jSLE. Delayed puberty was defined as delay in development of secondary sexual characteristics of more than 2SD below the mean for age by Tanner staging [10,12]. All medications utilized were also recorded.

Statistical analysis was done using the statistical package for social science (SPSS), version 14. Nominal data were expressed as frequency and percentage and were compared using the chi square test. Numerical data were expressed as range, mean and standard deviation and were compared using t tests. P values less than 0.05 were considered significant.

Ethics approval from the Research Ethics Committee, Faculty of Medicine, Cairo University, was not necessary according to University and Research Ethics Committee guidelines as all data and laboratory tests were obtained from patients' records, and as a part of their routine follow-up. All patient data was kept confidential.

## Results and discussion

The study included 148 patients; 103 females (69.6%) and 45 males (30.4%). The female to male ratio was 2.3:1. The mean age of diagnosis of jSLE was 10.5 ± 2.75 years (range 2-16 years). The mean disease duration was 6.57 ± 3.59 years.

The mean age at study visit was 17.1 ± 3.8 years. At presentation, constitutional manifestations were present in (44.6%), followed by mucocutaneous (27.7%), arthritis (27.7%), renal (21.6%), hematologic (10.8%), and NSP problems(10.1%).

The clinical and laboratory characteristics of these patients throughout the disease course were summarized in Table 1. The number of diagnostic criteria present in patients at disease presentation are summarized in Table 2.

**Table 1:** Clinical and laboratory characteristics of 148 patients with juvenile-onset systemic lupus erythematosus

Manifestation	(N.)percentage
Constitutional	(85) 58.1%

Mucocutaneous	(113) 76.4%

Musculoskeletal	(77) 52.7%

Hematological	(81) 55.4%

Renal	(96) 65.5%

Neuropsychiatric	(20) 31.8%

Gastrointestinal	(11) 8.1%

Cardiovascular	(59) 39.9%

Pulmonary	(31) 21.6%

**laboratory features**	

Hemolytic anemia	(40) 27.6%

Leucopenia	(54) 36.8%

Thrombocytopenia	(45) 30.9%

ANA	(139) 94.1%

Anti-ds DNA	(65) 44.1%

N.: number

**Table 2:** the number of diagnostic criteria present in 148 patients at time of presentation

Number of criteria at diagnosis	Percent of patients
2	5.4%

3	16.9%

4	37.7%

5	20.8%

6	13.8%

7	4.6%

8	0.8%

All patients were assessed

Disease activity and cumulative organ damage in the patients were assessed simultaneously at the study visit, as shown in Table 3. Overall, 43.9% patients had damage, with at least one item in the SDI (SDI 1), with a mean time of 6.57 ± 3.59 years from disease onset to the time of assessment. The mean SDI score was 0.93 ± 1.37 (range 0 - 6). The frequency of damage by organ system is shown in Table 4 with comparative results of 2 other similar studies. The organ systems most frequently affected by damage were the NSP (21%), renal (16.9%), cardiovascular (11.5%), skin (9.5%), ocular (6.1%), pulmonary (6.1%) and musculoskeletal (3.4%) systems. Only 2 patients (1.4%) developed diabetes and no malignancies were reported. The frequency of growth failure was 28.3%, while delayed puberty was present in 15.1% of our patients.

**Table 3:** Results of disease activity and organ damage assessments

SLEDAI	12.88	± 7.31
**SDI**	0.93	± 1.37

SLEDAI = Systemic Lupus Erythematosus Disease Activity Index (score range 0-105); SDI = Systemic Lupus International Collaborating Clinics/American College of Rheumatology Damage Index (score range 0-49).

**Table 4:** Frequency of damage in the 12 organ systems/domains and the 41 items of the SDI, in our study group, with comparison with the 2 other cohorts

Item	Percentage For our Egyptian study group 2012	Percentage **For Brunner HI et al, pediatric study group 2008 **[24]	**Percentage for Gutiérrez etal, study group 2006 **[20]
**Ocular**	**6.1%**	**42.4%**	**8.2%**

Cataract	3.4%	42.4	

Retinal change or optic atrophy	2.7%		

**Neuropsychiatric**	**21%**	**12.1%**	**10.7%**

Cognitive impairment or major psychosis	8.8%		

Seizures requiring therapy for 6 months	9.5%		

Cerebrovascular accident	2.7%		

Cranial or peripheral neuropathy (excluding optic)	0%		

Transverse myelitis	0%		

**Renal**	***16.9%***	***9.1%***	**13%**

Estimated or measured glomerular filtration rate < 50%	5.4%		

Proteinuria ≥ 3.5 gm/24 hours	6.1%		

End-stage renal disease	5.4%		

**Pulmonary**	**6.1%**	**3%**	**1.8%**

Pulmonary hypertension	4.7%		

Pulmonary fibrosis	1.4%		

Shrinking lung	0%		

Pleural fibrosis	0%		

Pulmonary infarction or lung resection not for malignancy	0%		

**Cardiovascular**	**11.5%**	**1.5%**	**3%**

Angina or coronary artery bypass	0%		

Myocardial infarction	0%		

Cardiomyopathy	2%		

Valvular disease	7.4%		

Pericarditis for 6 months or pericardiectomy	3.4%		

**Peripheral vascular**	**(4.8%)**	**3%**	**4.8%**

Claudication for 6 months	0%		

Minor tissue loss	1.4%		

Significant tissue loss	1.4%		

Venous thrombosis with swelling ulceration or venous stasis	2%		

**Gastrointestinal**	**0.7%**	**3%**	**2.2%**

Infarction or resection of bowel, spleen, liver, or gall bladder	0.7%		

Mesenteric insufficiency	0%		

Chronic peritonitis	0.7%		

Stricture or upper gastrointestinal tract surgery	0%		

Pancreatic insufficiency requiring enzyme replacement or with pseudo cyst	0%		

**Musculoskeletal**	**3.4%**	**16%**	**10.7%**

Muscle atrophy or weakness	0%		

Deforming or erosive arthritis	0%		

Osteoporosis with fracture or vertebral collapse	1.4%		

Avascular necrosis	1.4%		

Osteomyelitis	0.7%		

Ruptured tendons	0%		

**Skin**	**9.5%**	**5%**	**6.7%**

Scarring chronic alopecia	6.1%		

Extensive scarring or panniculum other than scalp and pulp space	1.4%		

Skin ulceration (excluding thrombosis) for more than 6 months	2%		

**Premature gonadal failure**	**0%**		**2.9%**

**Diabetes**	**1.4%**	**3%**	**0.4%**

**Malignancy**	**0%**	**0%**	**0%**

**Growth failure**	**28.3%**		**15.3%**

**Delayed puberty**	**15.1%**		**11.3%**

SDI = Systemic Lupus International Collaborating Clinics/American College of Rheumatology Damage Index

N.B: One patient may have more than one damage criteria

**Figure 1:**
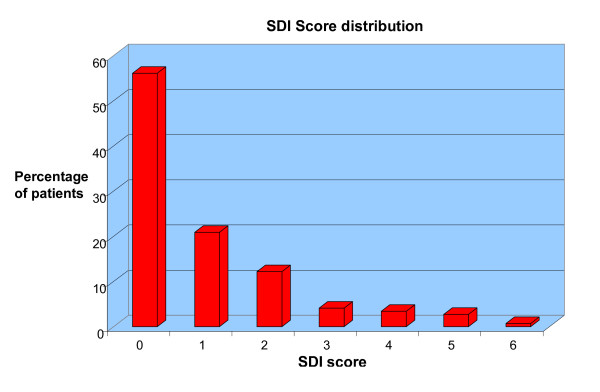
**showing distribution of patients according to SDI score**.

The distribution of patients according to SDI score is shown in Figure 1. The summary of drug therapies utilized by the patients throughout the disease course is reported in Table 5. Statistical analyses were conducted by comparing variables in patients classified according to the presence or absence of damage (i.e., patients with SDI ≥ 1 versus patients with SDI = 0).

**Table 5:** Summary of drug therapies

Medication	% of patients who received the therapy
Antimalarial medications, either daily or 3 times weekly	**83.0%**

Oral prednisone, either daily or alternate day	**97.8%**

Intravenous methylprednisolone pulses (30 mg/kg) at any time during disease course	**67.7%**

Cyclophosphamide pulses (500-1000 mg/m^2^)	**42%**

Azathioprine	**45%**

Mycofenolate mofetil	**8%**

Cyclosporine	**3%**

There was a significant correlation between the damage index and the total number of diagnostic criteria at presentation (p value = .007 and r = .236). Thus, an increased number of diagnostic criteria at presentation may increase the chance of developing more organ damage over time.

The presence of damage was also associated with the presence of the NPS ACR criterion (neuropsychiatric manifestations by American College of Rheumatology SLE criteria) at the time of SLE diagnosis (p = .018), i.e. patients with the NPS will have an increased chance of developing damage. Linear regression analysis was done for predictors of damage as detected by univariate analysis, again showing significance with the number of diagnostic criteria at presentation (p = 0.042) and NPS damage (p = 0.014).

We found that the renal damage was more prevalent in the group below 10 years of age at onset compared to children who presented at age greater than 10 years of age (p = .002). The mean disease duration of patients with musculoskeletal damage as assessed by the musculoskeletal (MSK) damage item in the SDI was significantly higher than in those without MSK damage (p = .0450). Thus it appeared that in our jSLE patients, the longer the jSLE disease duration, the more likely musculoskeletal damage will occur. The correlations between the SDI and drug therapies were not assessed as the total dose of each medication, as well as the duration of use, could not be assessed precisely for all patients. No association was found between disease activity assessed by SLEDAI and jSLE organ damage at the study visit.

Systemic lupus erythematosus is an autoimmune disease in which children and adolescents represent 15-20% of all patients [13]. Several studies have reported a wide range of variation in the natural history of jSLE among different ethnic and geographical groups [14], including studies among Egyptian children [15].

As a result of marked improvement in the prognosis of jSLE [5] with decreased mortality, more attention is now paid to morbidity as an indicator of outcome, as measured in terms of organ dysfunction resulting from the disease and its treatment [16,17]

In this work we studied the cumulative organ damage, as measured by the SDI, in 148 patients with jSLE with a mean disease duration of 6.57 ± 3.59 years.

Demographic, clinical and laboratory features of our patients are comparable to the jSLE features that have been reported in many other studies conducted in jSLE patients [6,18] and to studies among Egyptian children [15,19]

Significant cumulative damage, with at least one item in the SDI (SD ≥ 1), was present in 43.9% of our children, with the mean SDI score of 0.93 ± 1.37 (range 0 - 6). This is considerably lower than the damage in some other pediatric series which ranged from 61%, to 50.5% [6,20], but more than the percentage of damage reported in the study of Gutiérrez-Suárez et al., in which damage was detected in 39.9% of patients [21]. However, this difference in our study group may be due to an earlier age of disease diagnosis (mean 10.5 ± 2.75 years) and longer disease duration (mean 6.57 ± 3.593 years), when compared to those in the study of Gutiérrez-Suárez et al.

In our series, neuropsychiatric (NPS) damage was the most frequent type of damage, occurring in 21% of our patients. This is less than the percentage of neuropsychiatric damage reported by Lilleby et al. (28%) [20]. On the other hand, neuropsychiatric damage was higher than many other series. In these other studies the neuropsychiatric damage ranged from 15.8% [5] to 10.7% [21]. The higher percentage of neuropsychiatric damage in our series is mainly in the form of seizures, persisting more than 6 months, or cognitive impairment. Cognitive impairment was assessed in our patients only on a clinical basis, without using specific neurocognitive testing, and without any comparisons with previous cognitive assessments done to these patients.

Whether this clinical assessment without testing affects the results or not is unclear. In our study, renal damage was the second most common type of damage, occurring in 16.9% of the children. This high percentage of renal damage may be due to a high percentage of renal involvement in juvenile SLE in Egyptian children with renal disease as high as 65.5%, with relatively high percentage of Class III and Class IV nephritis, occurring in 25% and 22.5% of children and adolescents with jSLE, respectively [19].

Renal damage in our study was slightly less than that reported by some series (21.8%) [5] and higher than those in other series [20,21] where renal damage was detected in only 13% of patients.

Moreover, cardiovascular damage occurred in 11.5% of our children, of which 7.4% were due to valvular disease, as detected by the presence of diastolic or systolic murmur of a minimum grade of 3/6. However, due to the high prevalence of rheumatic fever heart disease in Egypt [22], further investigations regarding the possibility that some of these murmurs may be due to previously undiagnosed rheumatic fever carditis may be prudent. Skin damage was detected in 9.5% of our patients, with a scarring chronic alopecia being the commonest manifestation in 6.1%. This result is consistent with the study of Ravelli et al. 2005 (9.6%), but higher than that reported by Gutiérrez-Suárez et al. (7.6%) [21].

Although all investigators agreed that the estimation of SDI was appropriate for patients with jSLE, assessment of growth and pubertal age were also conducted for better assessment of jSLE damage for children and adolescents. The frequency of growth failure in our study group, was 28.3%, which is much higher than that reported by Gutiérrez-Suárez et al.(15.1%) [21]. This high rate of growth failure may be partly related to prolonged corticosteroid use and partly due to poor nutritional status of some of our children of low socioeconomic background. Disease activity may also play a role.

Delayed puberty was detected in 15.1% of our patients when assessed at time of assessment of damage. This percentage of puberty delay is slightly higher than the results of Gutiérrez-Suárez et al. (11.3%) [[20]]. This delayed puberty is a very important form of damage affecting the adolescent growth spurt of patients and sexual development and often leads to emotional and social difficulties.

When compared to adult series, the mean SDI score and the frequency of cumulative damage recorded in our patients were in the low range, with the mean SDI score being 1.51 ± 1.97 and the frequency of damage being 60%. For example, the higher percentage of damage in an adult series were obvious with frequencies such as musculoskeletal damage in 22%, ocular damage in 13%, and diabetes mellitus reported in 6% of adult patients [1]. This higher rate of damage in adults may be related to cumulative and high dose prednisone or the presence of other risk factors as smoking, obesity and atherosclerosis [1]. Renal damage was more prevalent in patients with younger disease presentation and with longer disease duration, which agrees with other pediatric and adult series [23,24].

No association was found between SLEDAI and SDI, which is expected as SDI describes accumulated damage over a period of time, whereas, SLEDAI is as assessment of disease activity at a certain time. The best way to assess the burden of the ongoing jSLE disease activity may be by assessing SLEDAI scores over time and by the area under the ROC curve. We acknowledge that one of the limitations of this work is our assessment of disease activity by the SLEDAI at one point of time only and that approach prevents such burden assessment.

The presence of damage was more prevalent in patients having a higher number of diagnostic criteria at disease presentation, as well as in those with neuropsychiatric manifestations at presentation, which is in concordance with other pediatric series [6].

Therefore, in these patients, proper management and follow-up are of great importance due to higher risk of development of permanent organ damage [6].

Assessment of the cumulative drug doses in our patients and its correlation with damage was not possible due to the large number of patients and lack of accurate recordings for all patients, especially with children with long disease durations. This is a significant limitation of our study as we could not correlate treatment with organ damage as done by Lilleby et al [20].

## Conclusions

The evidence of cumulative organ damage, as measured by the SDI, was found in 43.9% of our jSLE patients, and was not related to disease activity assessed by SLEDAI. Damage was significantly more likely in patients with longer disease duration and NPS manifestations at the time of diagnosis. These high risk groups require a strict management plan and follow-up. Growth retardation and delayed puberty are important items of jSLE damage that should be assessed in children, and attention should be paid to their prevention.

## Competing interests

The authors declare that they have no competing interests.

## Authors' contributions

**SS: **head of the pediatric rheumatology department Cairo University; participated in study conceptualization, supervision of the work steps, and critical revision of the manuscript. **HL **participated in data collection, statistical analysis of the results, writing the manuscript, and publication correspondence. **AN **participated in study conceptualization, data collection, interpretation of the results and final approval of the manuscript. **AMK **participated in statistical analysis of the results, helped in writing the manuscript and supervision of the work steps. **NF s**hared in data collection, tabulation of data, and final approval of the Manuscript. All authors read and approved the final manuscript.
